# Resolution of post-anaesthesia aortic regurgitation by repair of a ruptured sinus of Valsalva aneurysm: 5 case reports

**DOI:** 10.1186/s12872-020-01761-1

**Published:** 2020-11-03

**Authors:** Qian Xue, Chun Yang, Xiu Han

**Affiliations:** Department of Cardiology, First Affiliated Hospital of Xi’an Jiaotong University, Xi’an, 710061 Shaanxi China

**Keywords:** Post-anaesthesia aortic regurgitation, Ruptured sinus of Valsalva aneurysm, TEE

## Abstract

**Background:**

Ruptured sinus of Valsalva aneurysm (SVA) is a rare cardiovascular disease in which some patients exhibit aortic valve insufficiency. SVA repair and valve replacement are usually required for treatment. Here, we report 5 cases of ruptured SVA with severe post-anaesthesia aortic regurgitation (AR). To the best of our knowledge, this is the first report of ruptured SVA with severe post-anaesthesia AR.

Case presentation:

From 2018 to 2020, there were 5 cases of ruptured SVA with severe AR after anaesthesia in our hospital. The main symptoms were palpitation and shortness of breath. Transthoracic echocardiography (TTE) with colour-flow Doppler showed ruptured aortic sinus aneurysms without AR. Post-anaesthesia echocardiography showed severe AR. Direct patch closure of the ruptured aneurysm resolved the left-to-right shunt and AR, and the aortic valve was not replaced.

**Conclusions:**

Post-anaesthesia AR without obvious structural defects may occur in patients with ruptured SVAs. Valve replacement may not be necessary.

## Background

Sinus of Valsalva aneurysm (SVA) is a rare cardiovascular disease. In 1839, Hope gave the first report of a ruptured SVA, which occurred in a 25-year-old patient [1]. SVA occurs most frequently in the right sinus of Valsalva, followed by the noncoronary sinus; the least frequent location is the left coronary sinus [2]. The present study includes 5 patients with ruptured SVAs who experienced severe aortic regurgitation (AR) after anaesthesia. Simple patch closure of the ruptured aneurysm solved the left-to-right shunt and the AR. We discuss the causes of this phenomenon and propose that, for this type of patient, the surgeon needs to decide whether it is necessary to replace the aortic valve to eliminate aortic regurgitation based on the results of pre-anaesthesia echocardiography and an evaluation of the aortic valve structure.

## Case presentation

**Fig. 1:**
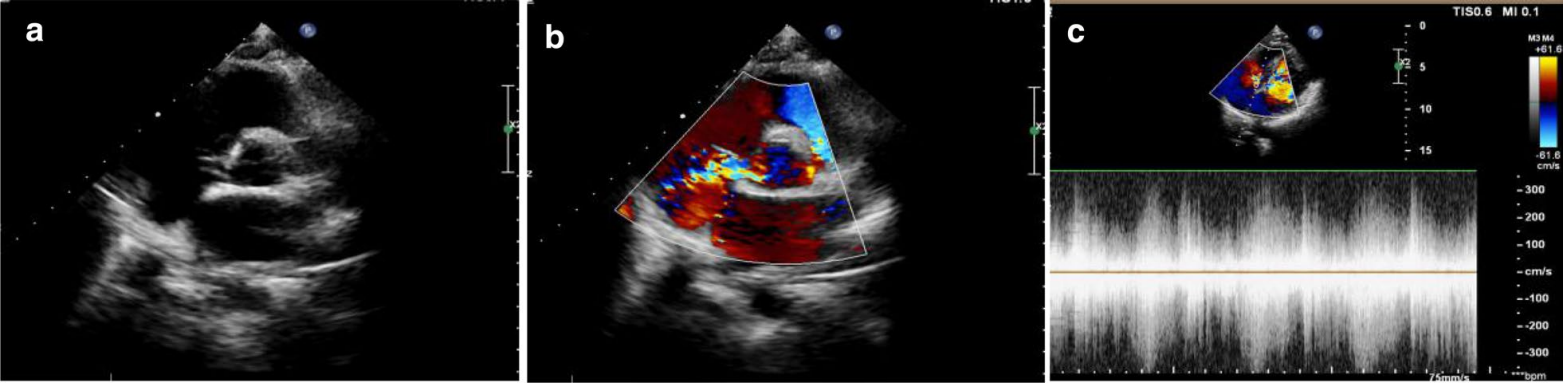
Transthoracic echocardiograms in one patient. **a** Two-dimensional and **b** colour-flow Doppler modes show the rupture of the noncoronary sinus into the right atrium. **c** Continuous-wave spectral Doppler mode shows continuous flow through the ruptured aneurysm

From 2018 to 2020, there were 5 cases of ruptured SVA with severe post-anaesthesia AR in our hospital. The 5 included patients ranged from 25 to 42 years of age, and all of them were male. Three of them had a history of hypertension. The time from onset to treatment varied from 7 to 45 days. The patients came to the hospital for treatment due to palpitations and shortness of breath. No treatment was given before admission. Cardiac auscultation revealed the presence of systolic and diastolic murmurs. Transthoracic echocardiography (TTE) with colour-flow Doppler showed ruptured aortic sinus aneurysms without AR. Four patients had a noncoronary sinus that had ruptured into the right atrium, and 1 patient had a right sinus of Valsalva that had ruptured into the right ventricle (Fig. 1).

**Fig. 2:**
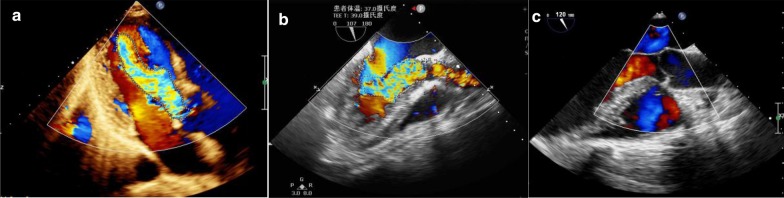
**a** Post-anaesthetic transthoracic echocardiography and **b** transoesophageal echocardiography showed severe aortic regurgitation. **c** Post-operative transoesophageal echocardiography showed no significant aortic regurgitation

All 5 patients were immediately admitted to the hospital for surgery. After anaesthesia, TTE and transoesophageal echocardiography (TEE) showed aortic insufficiency with severe regurgitation (Fig. 2). During the operation, the surgeon observed that the structure of the aortic valve and aortic root was basically normal; for example, the diameter of the aortic annulus was within the normal range (Table 1). Because preoperative TTE showed no AR, we decided to repair the ruptured SVA without valve replacement and observe the change in valve regurgitation. The SVA was repaired with autologous pericardium during cardiopulmonary bypass (CPB). After termination of the CPB and recovery of the heartbeat, TEE showed that the left-to-right shunt and AR were resolved. The patients were in good condition at the 1-month follow-up. Three months after discharge from the hospital, TTE confirmed that there was no AR.

**Table 1:** Post-anaesthetic TTE: cardiac structure and function

Case	Case 1	Case 2	Case 3	Case 4	Case 5
Age (years)	33	28	40	25	42
Location of the SVA	Noncoronary sinus	Noncoronary sinus	Noncoronary sinus	Noncoronary sinus	Right coronary sinus
Diameter of the base of the SVA (mm)	7	9	8	6	7
Diameter of the aortic sinus (mm)	28	31	30	29	30
Diameter of aortic annulus (mm)	23	25	25	24	25
Left ventricular end diastolic diameter (mm)	50	54	52	49	51
Left ventricular ejection fraction (%)	64	61	66	70	69
Area of AR after anaesthesia (cm^2^)	11.9	11.3	10.8	9.9	10.6

## Discussion

Most SVAs are congenital malformations, with very few of them being acquired. Congenital SVA refers to disease caused by congenital developmental defects of the sinus of Valsalva and a lack of normal elastic and muscle tissue. Under the continuous effect of the internal pressure of the aorta, the wall of the sinus gradually thins, and the sinus expands outward into an aneurysm that resembles a tumour. If rupture occurs, the structure is called a ruptured SVA. Acquired SVAs may be caused by diseases, e.g., infective endocarditis, syphilis, Marfan's syndrome, arteriosclerosis, trauma, and aortic dissection aneurysm. Most patients with ruptured SVAs have other cardiovascular malformations, e.g., ventricular septal defects, bicuspid aortic valves, aortic valve prolapse, pulmonary valve stenosis or aortic coarctation [2, 3].

SVA rupture is an acute and serious event. The larger the rupture is, the greater the pressure difference that may occur between the aorta and the ruptured chamber; additionally, the larger the shunt flow, the higher the haemodynamic impact that may result. Serious acute haemodynamic disturbance can quickly cause acute heart failure. Thus, urgent repair is necessary to reverse acute heart failure. Additionally, in some patients, sinus expansion and rupture can result in structural changes in the aortic root, aortic valve dislocation or prolapse, and valve dysfunction. Moreover, severe regurgitation will further aggravate the left ventricular volume load. Valve replacement is usually required for treatment.

Notably, no obvious AR was found in any of the 5 patients before the operation. The diameters of the aortic sinus and aortic annulus were normal. The structure of the aortic valve was basically normal. After anaesthesia, TEE showed aortic insufficiency and severe AR. After simple repair of the SVA defect, the regurgitation disappeared.

We propose the following reasons for the occurrence of AR after anaesthesia and for the disappearance of AR after repair of the ruptured SVA. A ruptured SVA causes destruction of the integrity of the sinus wall and weakening of its supportive function, leading to changes in the spatial structure of the aortic root and abnormal closure of the valve. At the same time, the blood flow from the ruptured SVA produces a low-pressure zone (Venturi effect) [4]. Because of this pressure difference, the aortic valve does not prolapse significantly, and thus, there is no AR before the operation. However, such balance can be broken after anaesthesia. Anaesthetics cause vasodilation and decrease the myocardial contractile force [5–7], leading to a decrease in blood pressure, a decrease in shunt flow, a decrease in the Venturi effect and, ultimately, aortic valve prolapse and insufficiency. After repair of the ruptured SVA, the integrity of the sinus is restored, valve closure returns to normal, and regurgitation without obvious structural defects in the aortic valve disappears.

Not all patients with ruptured SVA share this phenomenon; at present, we are aware of only these five. We found that aortic valve prolapse may be due to congenital dysplasia of the aortic sinus and an absence of aortic media behind the aortic annulus [8]. The diameter of the base of the SVA may also be related. The larger the diameter of the base of the SVA, the worse the integrity of the aortic sinus is, and the less support the wall of the aortic sinus will provide. After anaesthesia, the pressure difference of the ruptured SVA is reduced, and the aneurysm becomes more likely to collapse, which will result in a structural change in the aortic root, leading to aortic valve prolapse. The detailed pathophysiological mechanism needs to be further verified by relevant studies.

## Conclusions

Ruptured SVAs are a rare and acute event with high mortality, and they require urgent repair. Some such patients also have aortic insufficiency, which may lead to further aggravation of left ventricular overload and heart failure. Valve replacement may be necessary for these patients. However, post-anaesthesia AR without obvious structural defects may occur in patients with ruptured SVAs. Valve replacement may not be necessary.

## Data Availability

All the data supporting our findings are contained within the manuscript.

## Abbreviations

SVA: Sinus of Valsalva aneurysm
AR: Aortic regurgitation
TTE: Transthoracic echocardiography
TEE: Transoesophageal echocardiography
CPB: Cardiopulmonary bypass

## References

[1] Hope J. A treatise on the diseases of the heart and great vessels:and on the affections which may be mistaken for them: comprising the author’s view of the physiology of the heart’s actions and sounds, as demonstrated by his experiments on the motions and sounds in 1830, and on the sounds in 1834-35. 3rd ed. London: John Churchill; 1839. p. 466–71.

[2] Takach TJ, Reul GJ, Duncan JM (1999). Sinus of Valsalva aneurysm or fistula: management and outcome. Ann Thorac Surg.

[3] Liu YL, Xiong JR (2014). Clinical echocardiography.

[4] Nascimbene A, Joggerst S, Reddy KJ (2013). Aortic valve regurgitation that resolved: after a ruptured coronary sinus aneurysm was patched. Tex Heart Inst J.

[5] Vuyk J, Sitsen E, Reekers M, Miller RD, Cohen NH, Eriksson LI (2015). Intravenous anesthetics. Miller’s anesthesia.

[6] Muzi M, Berens RA, Kampine JP (1992). Venodilation contributes to propofol-mediated hypotension in humans. Anesth Analg.

[7] Robinson BJ, Ebert TJ, O’Brien TJ (1997). Mechanisms whereby propofol mediates peripheral vasodilation in humans Sympathoinhibition or direct vascular relaxation?. Anesthesiology.

[8] Sakakibara S, Konno S (1962). Congenital aneurysm of the sinus of valsalva anatomy and classification. Am Heart J.

